# A novel osteoporosis model with ascorbic acid deficiency in Akr1A1 gene knockout mice

**DOI:** 10.18632/oncotarget.14458

**Published:** 2017-01-02

**Authors:** Cheng-Wei Lai, Hsiao-Ling Chen, Min-Yu Tu, Wei-Yu Lin, Theresa Röhrig, Shang-Hsun Yang, Ying-Wei Lan, Kowit-Yu Chong, Chuan-Mu Chen

**Affiliations:** ^1^ Department of Life Sciences, National Chung Hsing University, Taichung, Taiwan; ^2^ Agricultural Biotechnology Center, National Chung Hsing University, Taichung, Taiwan; ^3^ Department of Bioresources, Da-Yeh University, Changhua, Taiwan; ^4^ Department of Orthopaedic Surgery, Taichung Armed Forces General Hospital, Taichung, Taiwan and National Defense Medical Center, Taipei, Taiwan; ^5^ Department of Biomedical Engineering, Hungkuang University, Taichung, Taiwan; ^6^ Department of Physiology and Institute of Basic Medical Sciences, National Cheng Kung University, Tainan, Taiwan; ^7^ Department of Medical Biotechnology and Laboratory Science, College of Medicine, Chang Gung University, Tao-Yuan, Taiwan; ^8^ Department of Thoracic Medicine, Chang Gung Memorial Hospital at Linkou, Tao-Yuan, Taiwan; ^9^ Rong-Hsing Translational Medicine Center, and iEGG Center, National Chung Hsing University, Taichung, Taiwan

**Keywords:** osteoporosis, Akr1A1 gene, ascorbic acid, knockout mice, micro-CT imaging, trabecular bone, cortical bone, Pathology Section

## Abstract

The AKR1A1 protein is a member of the aldo-keto reductase superfamily that is responsible for the conversion of D-glucuronate to L-gulonate in the ascorbic acid (vitamin C) synthesis pathway. In a *pCAG-eGFP* transgenic mouse line that was produced by pronuclear microinjection, the integration of the transgene resulted in a 30-kb genomic DNA deletion, including the *Akr1A1* gene, and thus caused the knockout (KO) of the *Akr1A1* gene and targeting of the *eGFP* gene. The *Akr1A1* KO mice (*Akr1A1*eGFP/eGFP) exhibited insufficient serum ascorbic acid levels, abnormal bone development and osteoporosis. Using micro-CT analysis, the results showed that the microarchitecture of the 12-week-old *Akr1A1*eGFP/eGFP mouse femur was shorter in length and exhibited less cortical bone thickness, enlargement of the bone marrow cavity and a complete loss of the trabecular bone in the distal femur. The femoral head and neck of the proximal femur also showed a severe loss of bone mass. Based on the decreased levels of serum osteocalcin and osteoblast activity in the *Akr1A1*eGFP/eGFP mice, the osteoporosis might be caused by impaired bone formation. In addition, administration of ascorbic acid to the *Akr1A1*eGFP/eGFP mice significantly prevented the condition of osteoporotic femurs and increased bone formation. Therefore, through ascorbic acid administration, the *Akr1A1* KO mice exhibited controllable osteoporosis and may serve as a novel model for osteoporotic research.

## INTRODUCTION

According to the National Institutes of Health (NIH) consensus statement in 2000, osteoporosis is defined as a skeletal disorder characterized by compromised bone strength, predisposing individuals to an increased risk of fracture [1]. The disease has become one of the major threats to public health due to the increased risk of bone fracture, including fracture of the hip and vertebrae, which severely increases disability and mortality [2]. However, the prevalence of osteoporosis is steadily increasing because of the demographic change in aging societies. In the United States, more than 10 million people are estimated to have osteoporosis, and more than 30% of the population that is over 50 years old in Taiwan is diagnosed with osteoporosis [3].

Bone mineral density (BMD), which is measured by dual-energy X-ray absorptiometry (DXA), is the predominant measurement that is used for the diagnosis of osteoporosis [4]. The World Health Organization (WHO) defines a diagnosis of osteoporosis as a peak bone mass (T-score) BMD of 2.5 standard deviations below the mean (T-score < -2.5), and a T-score between -1 to -2.5 is considered osteopenia [5]. Based on the causes, osteoporosis can be divided into primary and secondary osteoporosis [6,7]. Primary osteoporosis occurs mainly because of natural ageing. The BMD of men and women begins to decline after midlife, especially in postmenopausal women, who experience rapid bone loss in the early years of postmenopause. Two-thirds of osteoporotic fractures are observed in postmenopausal women [8], and, similarly, hypogonadism also increases bone loss in elderly men. In addition, secondary osteoporosis is usually caused by external factors, e.g., medications, endocrine, metabolism disorders and other conditions. However, osteoporosis can occur in all populations at all ages because of not only bone loss but also insufficient bone formation. Individuals who are deficient in nutrients (e.g., calcium, vitamin D and vitamin C), hormones (e.g., sex and growth hormones), and exercise, particularly during the critical bone formation period, may have a reduced peak bone mass throughout young adulthood and are consequently at an increased risk of osteoporosis [1].

Bone metabolism is a continuous process. The dynamics of bone formation and resorption, which are carried out by osteoblasts and osteoclasts, respectively, comprise the bone remodeling cycle [9, 10]. During bone formation, bone morphogenetic proteins (BMPs) activate osteoblasts via the transcription factors Dlx5, Runx2 and Osx. The activated osteoblasts then synthesize an abundance of collagen type I as the framework of bone structure and mineralize the bone via alkaline phosphatase (ALP), osteopontin, osteocalcin and 1,25-dihydroxyvitamin D3 [11, 12]. Clinical and nutritional studies showed that insufficient intake of ascorbic acid can reduce bone density and increase the risk of fractures in humans [13, 14]. The ascorbic acid synthesis pathway in many species, including mice and rats, involves 3 anabolic enzymes: glucuronate reductase (AKR1A1), which converts D-glucuronate to L-gulonate; gulonolactonase (SMP30), which converts L-gulonate to L-gulono-γ-lactone; and L-gulono-γ-lactone oxidase (GULO), which catalyzes the final step to produce L-ascorbic acid. However, unlike mice and rats, humans and other primates do not synthesize ascorbic acid due to a mutated and nonfunctional *Gulo* gene; therefore, the required ascorbic acid is only obtained from food.

In this study, we generated genetically modified mice that are unable to synthesize ascorbic acid due to a knockout (KO) of the ascorbic acid synthesis gene *Akr1A1*. The mice displayed a rapid induction of osteoporosis after ascorbic acid withdrawal, which could be rescued by the addition of ascorbic acid into the dietary water. Through ascorbic acid administration and micro-computed tomography (micro-CT) analysis, the *Akr1A1* knockout mice represent a controllable osteoporosis model that may allow for the mimicry of the desired features of human osteoporosis

## RESULTS

### The characterization of *Akr1A1* KO mice

**Figure 1:**
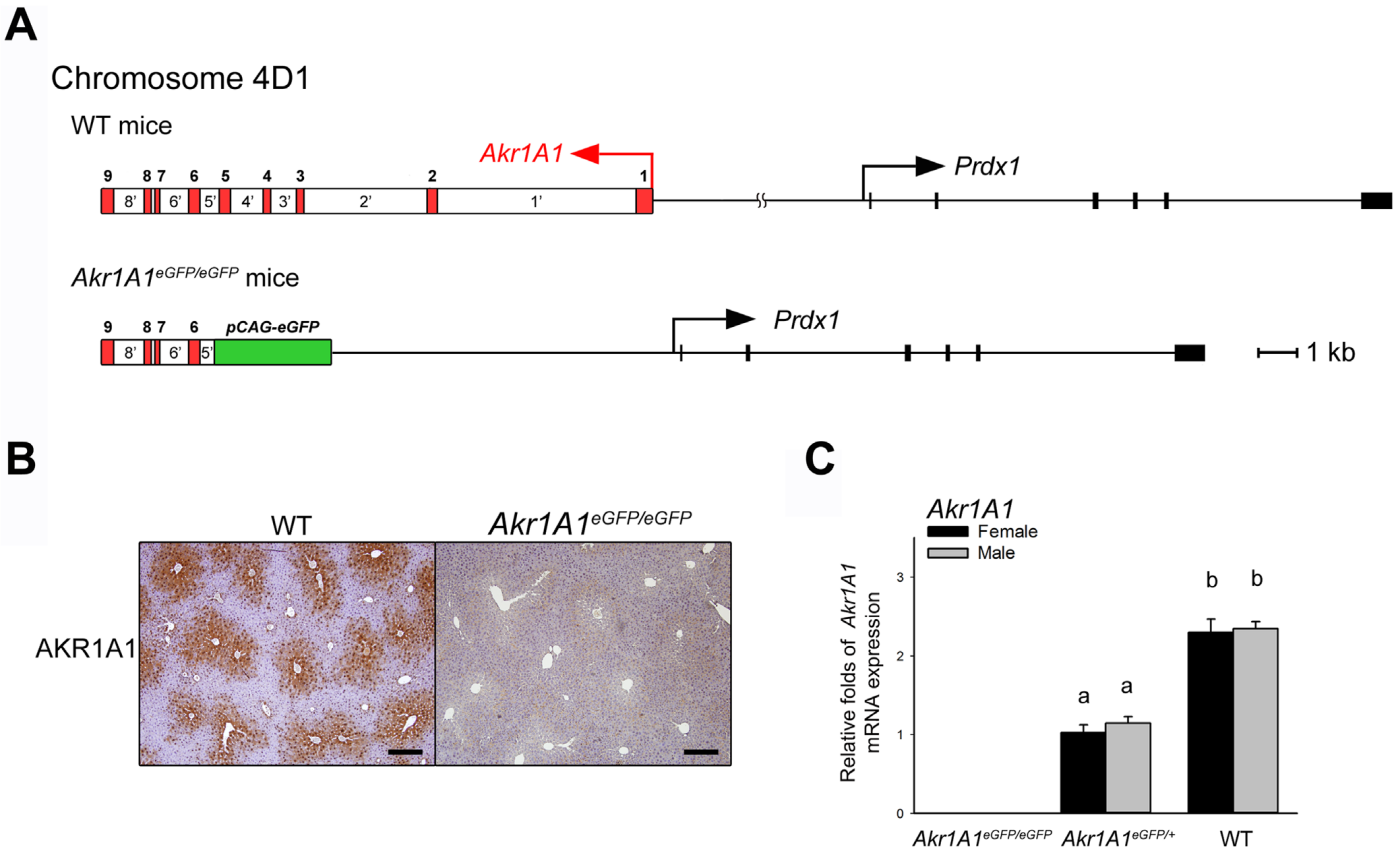
The characterization of the Akr1A1 KO mice **A**. The *Akr1A1* KO mice were produced by microinjection, which the integration of the *pCAG-eGFP* transgene (green) located in the chromosome 4D1 position, and caused the deletion of exon 1-5 (red), intron 1’-4’ (white) of the *Akr1A1* gene and the non-coding nucleotides that between *Akr1A1* and *Prdx1* genes. Scale bar: 1 kb. **B**. The IHC analysis of AKR1A1 protein expressions in WT and *Akr1A1*eGFP/eGFP mouse livers. The brown color indicated the AKR1A1 expressed hepatocytes. Scale bar: 400 μm. **C**. The *Akr1A1* mRNA expressions in *Akr1A1*eGFP/eGFP, *Akr1A1*eGFP/+ and WT mouse livers. The values are represented as the mean ± SE (*n* = 6); data were analyzed by the one-way ANOVA with Duncan's new multiple range test; the significant differences (*P* < 0.05) are represented with different letters in all columns.

The *pCAG-eGFP* transgenic mouse line was generated by pronuclear microinjection. Using chromosome FISH and CELI-PCR [15], the results demonstrated that the integration of the transgene gave rise to a 30-kb genomic DNA deletion that included exons 1-5 and introns 1-4 of the *Akr1A1* gene as well as the non-coding nucleotides between the *Akr1A1* and *Prdx1* genes, resulting in the generation of *Akr1A1* KO mice (Figure 1A). Previous studies showed that AKR1A1 is an NADPH-dependent aldehyde reductase that is expressed in the liver [16, 17]. In the *Akr1A1*eGFP/eGFP KO mice, *Akr1A1* mRNA and protein expression was abolished in the livers, and mRNA expression was reduced by 50% in *Akr1A1*eGFP/+ mice compared with wild-type (WT) mice (Figure 1B and 1C).

The body weight of *Akr1A1*eGFP/eGFP KO mice displayed a significant decrease when compared with WT mice (*P* < 0.01), but *Akr1A1*eGFP/eGFP + Vit. C treated group was able to restore the body weight to normal level (Supplementary Figure S1A). Furthermore, the results of liver pathological examination showed that *Akr1A1* gene knockout caused moderate severe aging of hepatocytes with intra nuclear inclusion body and glycogen accumulation in *Akr1A1*eGFP/eGFP KO mice (Supplementary Figure S1C) compared with WT mice (Supplementary Figure S1B) and *Akr1A1*eGFP/eGFP + Vit. C treated group (Supplementary Figure S1D).

### The *Akr1A1* KO mice exhibited ascorbic acid deficiency, increased ROS and osteoporosis which could be prevented by ascorbic acid supplementation

**Figure 2:**
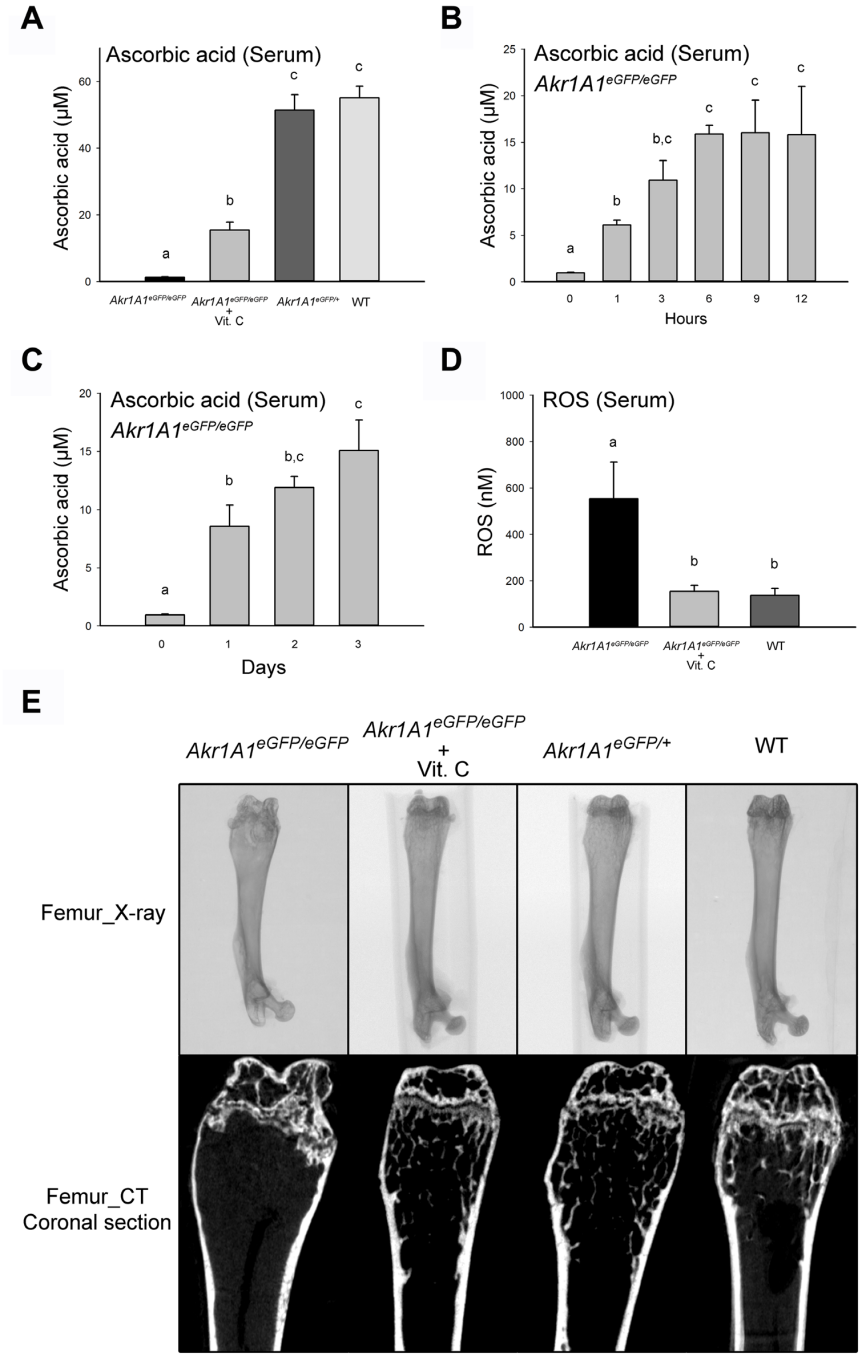
The absent of ascorbic acid, increased ROS and abnormal development of femurs in the Akr1A1 KO mice were prevented by ascorbic acid supplement **A**. The ascorbic acid concentration in the blood serum of *Akr1A1*eGFP/eGFP (*n* = 6), *Akr1A1*eGFP/eGFP with ascorbic acid supplement (350 mg/L) (n = 7), *Akr1A1*eGFP/+ (*n* = 6) and WT (n = 6) mice. **B**. and **C**. The ascorbic acid concentration of blood serum in *Akr1A1*eGFP/eGFP mice at different time points after the mice were fed with ascorbic acid (350 mg/L) in drinking water (*n* = 6). **D**. The ROS concentration in the serum of *Akr1A1*eGFP/eGFP (*n* = 6), *Akr1A1*eGFP/eGFP with ascorbic acid supplement (350 mg/L) (*n* = 7) and WT (*n* = 6) mice. The values are represented as the mean ± SE; data were analyzed by the one-way ANOVA with Duncan's new multiple range test. The bars with different letters represent *P* < 0.05. **E**. The X-ray images and coronal sections of micro-CT analysis from the femurs of *Akr1A1*eGFP/eGFP, *Akr1A1*eGFP/eGFP with ascorbic acid supplement (350 mg/L), *Akr1A1*eGFP/+ and WT mice.

The analysis of serum revealed that the concentration of ascorbic acid in *Akr1A1* eGFP/eGFP mice (1.25 ± 0.23 μM) was significantly lower than that in *Akr1A1*eGFP/+ (51.35 ± 8.14 μM) and WT (55.05 ± 7.73 μM) mice (*P* < 0.01), indicating that ascorbic acid synthesis was nearly abolished and resulting in insufficient ascorbic acid levels in the *Akr1A1* KO mice. However, there was no significant difference between the *Akr1A1*eGFP/+ and WT mice (Figure 2A). Additionally, after supplementation with freshly prepared ascorbic acid-containing water (350 mg/L), the serum ascorbic acid concentration increased in the *Akr1A1* eGFP/eGFP mice (15.38 ± 6.40 μM; *P* < 10^-4^ vs. *Akr1A1* eGFP/eGFP mice) (Figure 2A). The ascorbic acid level in the mouse serum began to increase after 1 hour of supplementation and reached a steady level after 6 hours, and the ascorbic acid levels of the serum were between 10~15 μM for at least 3 days (Figure 2B and 2C). Although the female *Akr1A1* KO mice were able to become pregnant and deliver their young, they could not successfully rear their neonates throughout the lactation period. When ascorbic acid was supplied to the *Akr1A1* KO mice in the drinking water, these female and male *Akr1A1* KO mice were able to breed directly with no differences in growth compared to WT mice. These results indicated that the ascorbic acid deficiency severely inhibited the bone development of the *Akr1A1* KO mice. In previous studies, low ascorbic acid intake will increase ROS production [18]. In the present study, the ROS level in the *Akr1A1* KO mouse serum was significantly increased compared with WT mice (*P* < 0.05) and returned to normal after supplementation with ascorbic acid (Figure 2D). These results suggested that the supplied ascorbic acid was sufficient to reduce the ROS level, even though the ascorbic acid level was only one quarter of that in the *Akr1A1*eGFP/+ and WT mice.

The risk of femoral fracture has been commonly used to evaluate osteoporosis, including the FDA guideline for evaluating the treatment of postmenopausal osteoporosis [19]. While rearing the *Akr1A1* KO mice, an abnormal walking posture was observed before the age of 6 months, and some of the mice became paralyzed at around 1 year of age. In the 12-week-old *Akr1A1* KO mice, the femurs shown abnormal structure where the distal femurs were enlarged and shortened. The coronal sections of the distal femurs in the *Akr1A1*eGFP/eGFP mice showed that the trabecular bones were severely reduced compared with the *Akr1A1*eGFP/eGFP + Vit. C, *Akr1A1*eGFP/+ and WT groups (Figure 2E). These results showed that the *Akr1A1* KO mice displayed abnormal bone development and severe osteoporosis even at a young age (12 weeks old).

### The micro-CT analysis of the trabecular and cortical bones in distal femurs

**Figure 3:**
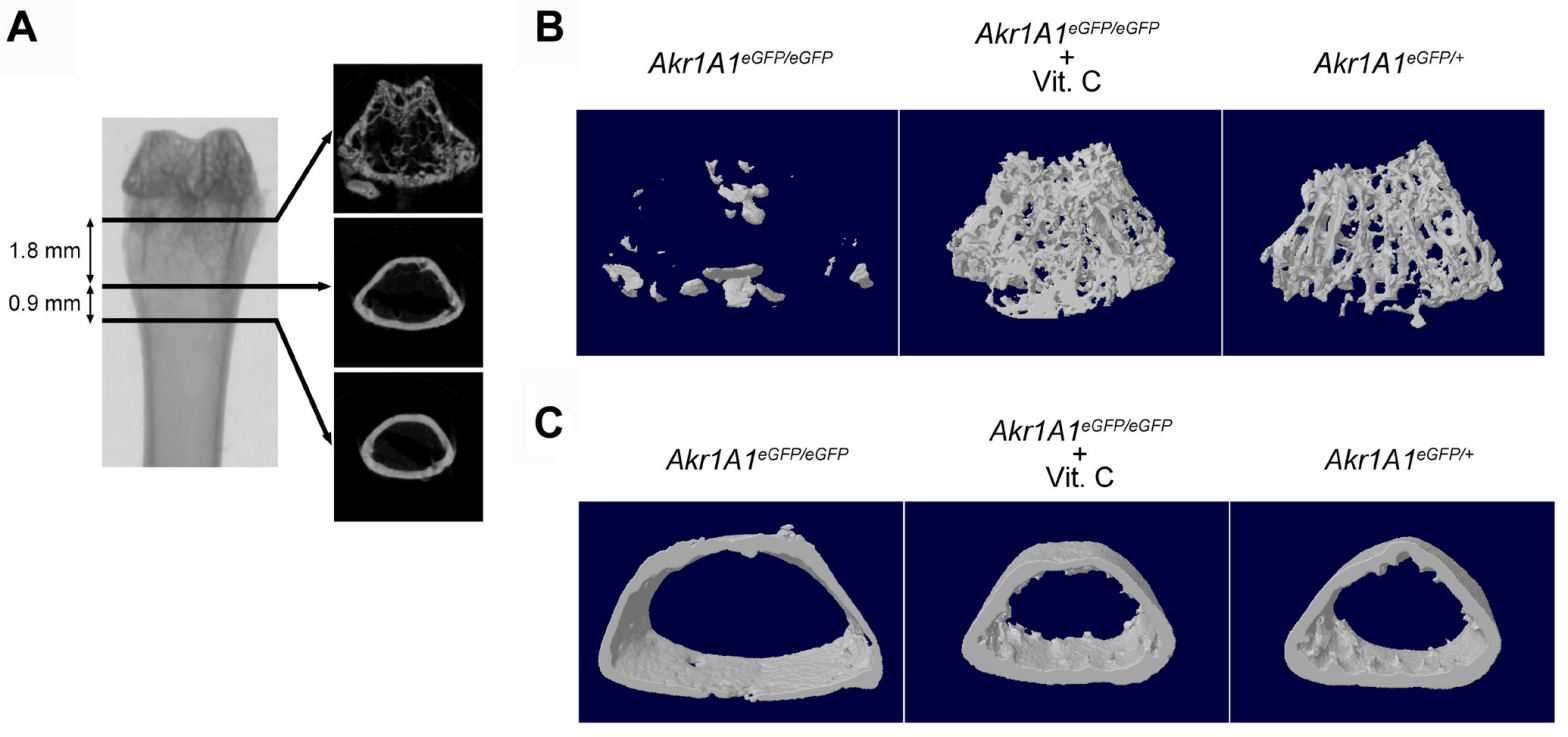
The 3D structure of trabecular and cortical bones in the distal femurs **A**. The micro-CT analyzed region of the femurs where the 1.8 and 0.9 mm regions were used to construct the 3D structure of trabecular and cortical bones, respectively. The femur sections showed the structures of upper and lower boundary of the analyzed regions. **B**. and **C**. The structures of trabecular and cortical bones in the distal femurs of *Akr1A1*eGFP/eGFP, *Akr1A1*eGFP/eGFP with ascorbic acid supplement and *Akr1A1*eGFP/+ mice.

To reveal the condition of the bone in *Akr1A1* KO mice, we used micro-CT to analyze and compare the microarchitecture of the femurs, which included the trabecular and cortical bone of the distal femur and the femoral head and neck of the proximal femur, among the *Akr1A1*eGFP/eGFP, *Akr1A1*eGFP/eGFP + Vit. C, *Akr1A1*eGFP/+ and WT groups. The region that was 1.8 mm thick from the end of the epiphysis plate contained the most trabecular bones in the distal femurs of the WT mice, and the region that was 0.9 mm thick from the end of the trabecular bone selected region was used to construct and analyze the 3D structure of the trabecular and cortical bones (Figure 3A). The 3D structure showed that the number of trabecular bones in *Akr1A1*eGFP/eGFP mice were severely reduced and that ascorbic acid supplementation could rescue trabecular bone formation (Figure 3B). Additionally, the 3D structure of the cortical bone revealed bone marrow cavity enlargement and cortical bone thinness in the *Akr1A1*eGFP/eGFP mouse femur compared with the other groups (Figure 3C).

**Figure 4:**
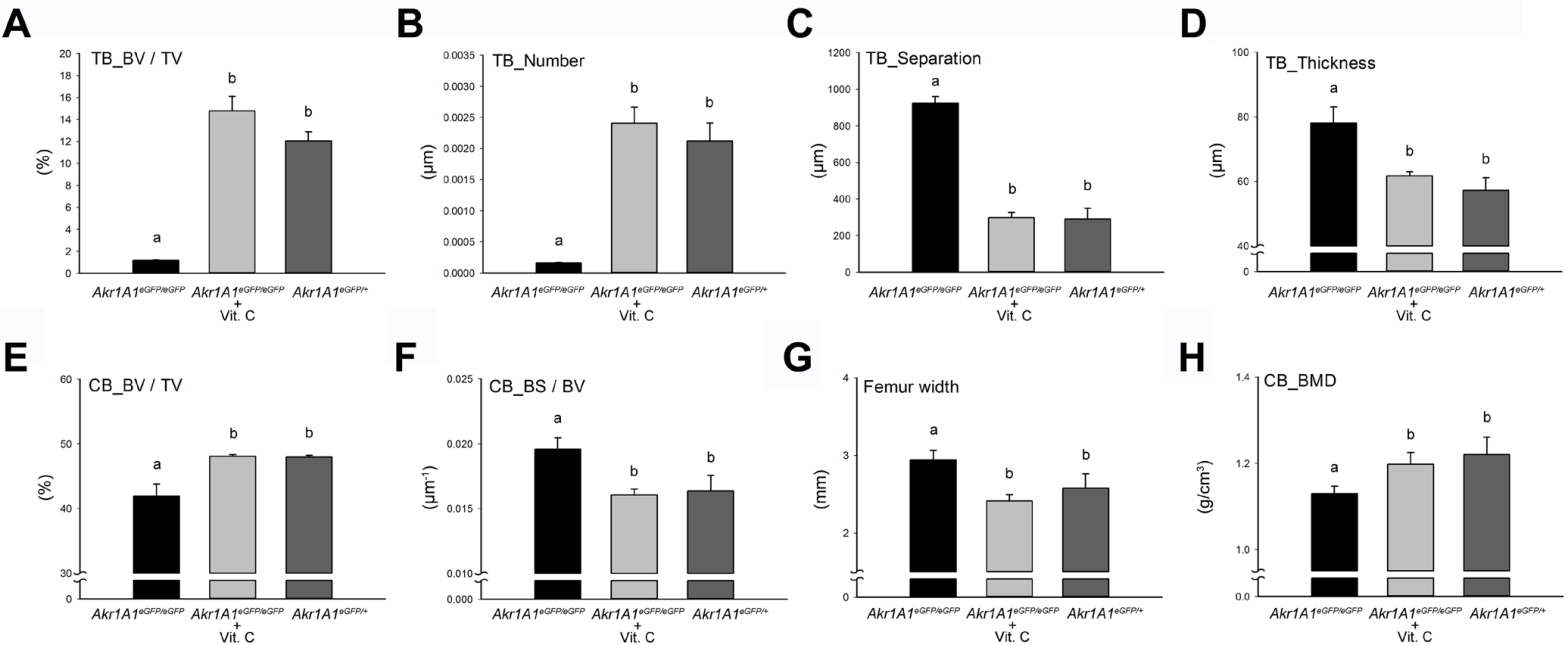
The micro-CT analytic scores of trabecular and cortical bones of the distal femurs The trabecular bone (TB) scores of bone volume (TB_BV/TV) **A**., trabecular number (Tb.N) **B**., trabecular separation (Tb.Sp) **C**. and trabecular thickness (Tb.Th) **D**., and the cortical bone (CB) scores of bone volume (CB_BV/TV) **E**., bone surface (CB_BS/BV) **F**., femoral width **G**. and bone mineral density (CB_BMD) **H**. in the regions, which were showed in Figure 3B and 3C, of *Akr1A1*eGFP/eGFP (*n* = 6), *Akr1A1*eGFP/eGFP with ascorbic acid supplement (*n* = 6) and *Akr1A1*eGFP/+ (*n* = 6) mouse femurs. The values are represented as the mean ± SE; data were analyzed by the one-way ANOVA with Duncan's new multiple range test. The bars with different letters represent *P* < 0.05.

The micro-CT analysis that included bone volume (BV) / tissue volume (TV) and the numbers of trabecular bones (Tb.N) displayed results similar to the 3D structures (Figure 4A and 4B). The measurements of trabecular bone separation (Tb.Sp) and thickness (Tb.Th) indicated that the spongy structures of the trabecular bone were not present in the *Akr1A1*eGFP/eGFP mice (Figure 4C and 4D) and only thick trabecular bones were distributed around the edge of the bone marrow cavity (Figure 3B). Additionally, the measurement of the cortical bone revealed that the proportion of cortical bone volume (BV / TV) was reduced (Figure 4E) and the bone surface (BS) / BV and the diameter of the femur (Figure 4F and 4H) were increased in the *Akr1A1*eGFP/eGFP mice compared with the other groups. These data demonstrated that the decreased bone volume and the increased surface area both resulted in increased weight loading per mm^2^ of the cortical bone in the *Akr1A1*eGFP/eGFP mice. In addition to the quantity of cortical bones, the quality of the cortical bone was also evaluated according to the BMD. The results showed that the BMD of cortical bones was also reduced in the *Akr1A1*eGFP/eGFP mice compared with the other groups (Figure 4G). These results indicated that reduced formation and strength of the trabecular bones in the femurs of the *Akr1A1*eGFP/eGFP mice compared with the *Akr1A1*eGFP/eGFP + Vit. C and *Akr1A1*eGFP/+ groups.

### The micro-CT analysis of proximal femurs

**Figure 5:**
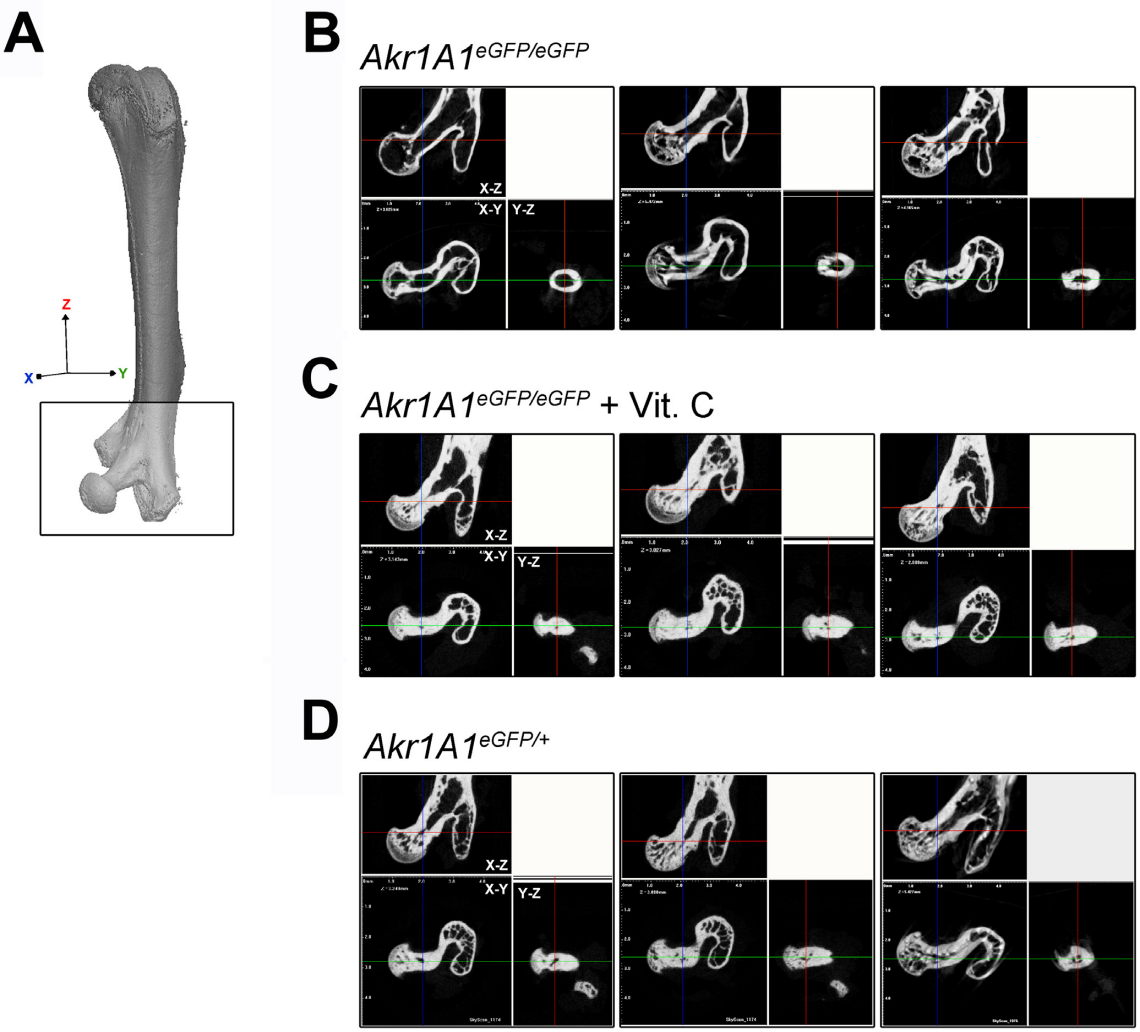
The structures of femoral head and neck at the proximal femurs **A**. The micro-CT analyzed region of femoral head and neck at the proximal femur (the square region). The X, Y, Z axis represented the orientation of the femur. **B**., **C**. and **D**. The sections of the X-Z, X-Y and Y-Z axial planes of the proximal femurs in *Akr1A1*eGFP/eGFP, *Akr1A1*eGFP/eGFP with ascorbic acid supplement and *Akr1A1*eGFP/+ mice, respectively, and the results were showed in triplicate.

Proximal femur fractures comprise nearly 20% of all osteoporotic fractures in humans. Therefore, the bone quality of the proximal femur, including the femoral head and neck, are important factors for evaluating osteoporosis. To examine the osteoporotic condition of femoral head and neck through micro-CT imaging, the femoral head and neck were aligned with the X-Z axial plane of the femur, and X-Z, Y-Z and X-Y sections were used to reveal the proximal femur structures (Figure 5A). The results showed that the bone mass in the femoral head and neck was reduced in the *Akr1A1*eGFP/eGFP mice and that the femoral neck cavity was enlarged and the trabecular structure was decreased in the femoral head compared with the other groups (Figure 5B, 5C and 5D).

### Osteoporosis in the *Akr1A1* KO mice was caused by insufficient bone formation

**Figure 6:**
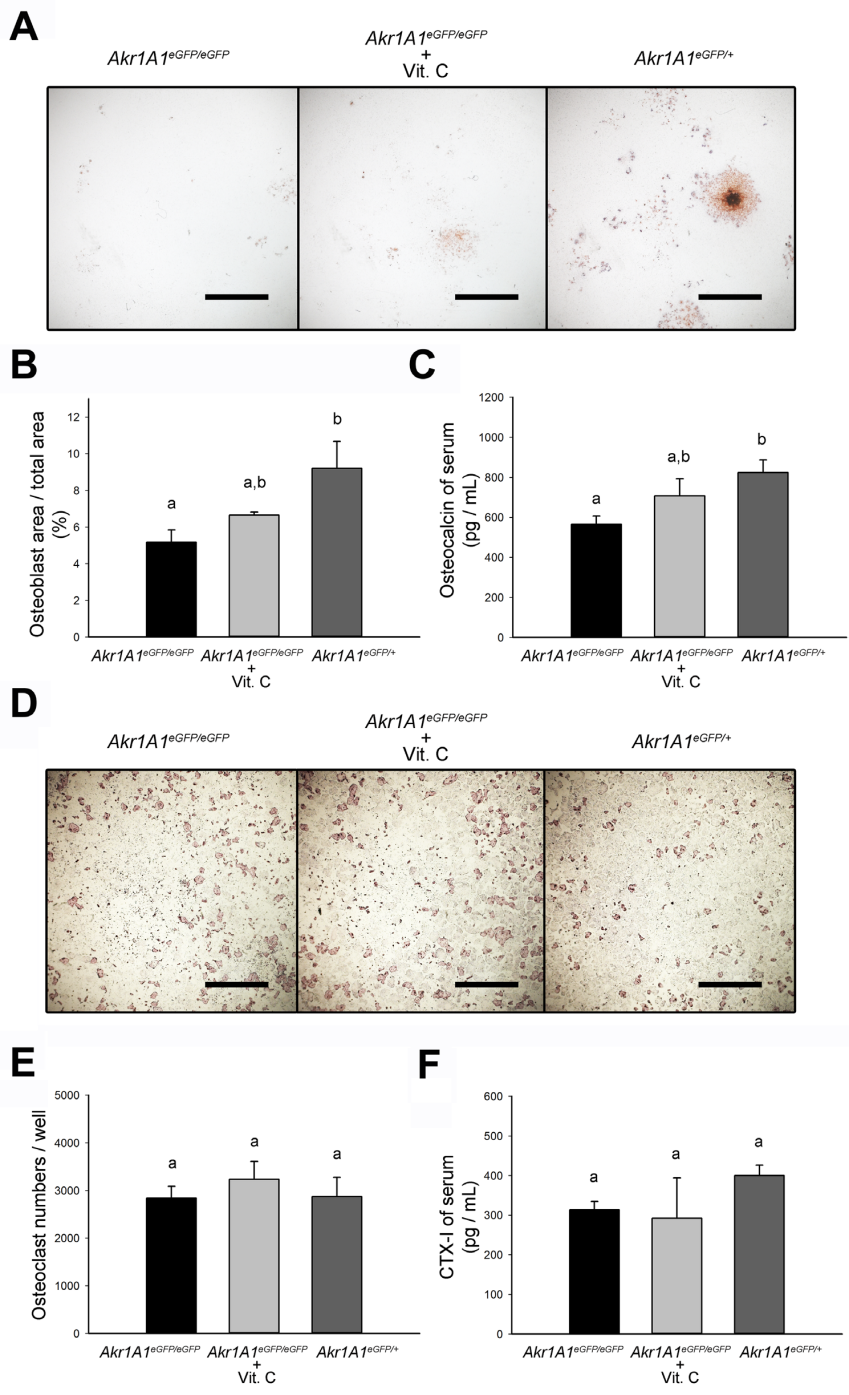
The decreased osteoblast number and bone formation in the Akr1A1 KO mice **A**. and **B**. The CFU-OB assay of *Akr1A1*eGFP/eGFP (*n* = 6), *Akr1A1*eGFP/eGFP with ascorbic acid supplement (*n* = 6) and *Akr1A1*eGFP/+ (*n* = 6) mouse femurs and the osteoblast area in 35 mm culture dishes. **C**. The quantification of bone formation marker, osteocalcin, in the groups. **D**. and **E**. The *in vitro* osteoclast formation assay of *Akr1A1*eGFP/eGFP (*n* = 6), *Akr1A1*eGFP/eGFP with ascorbic acid supplement (*n* = 6) and *Akr1A1*eGFP/+ (*n* = 6) mouse femurs and the osteoclast numbers in 35 mm culture dishes. **F**. The quantification of bone resorption marker, CTX-I, in the groups. Scale bar: 0.5 mm. The values are represented as the mean ± SE; data were analyzed by the one-way ANOVA with Duncan's new multiple range test. The bars with different letters represent *P* < 0.05.

The occurrence of most osteoporosis was due to imbalanced bone metabolism. To evaluate the causes of osteoporosis in the *Akr1A1* KO mice, the osteoblast and osteoclast activities and the osteoporosis markers in the blood serum were analyzed. Using a colony-forming unit-osteoblast (CFU-OB) assay, the active osteoblast area of the bone marrow cell culture was found to be reduced in the *Akr1A1*eGFP/eGFP group compared with the *Akr1A1*eGFP/+ group, and the area of the *Akr1A1*eGFP/eGFP + Vit. C group was slightly increased compared with that of the *Akr1A1*eGFP/eGFP group (Figure 6A and 6B). Osteocalcin is the most abundant non-collagenous protein in bone matrix. It is produced by osteoblasts and some osteocalcin is released into circulation during bone formation. Therefore, the serum osteocalcin level was used to evaluate the activity of bone formation [20]. The serum osteocalcin level in these three groups also showed similar results with that of the CFU-OB assay (Figure 6C). Additionally, using an *in vitro* osteoclast formation assay, the results showed that there was no significant difference in the number of active osteoclasts in the bone marrow cell culture among the three groups (Figure 6D and 6E); the C-terminal telopeptide of collagen I (CTX-I) of blood serum, which was used as a specific marker for the activity of bone resorption [21], also showed a similar result (Figure 6F). These results may imply that the activity of osteoblasts and bone formation rather osteoclasts and bone resorption were responsible for the osteoporosis in the *Akr1A1* KO mice.

## DISCUSSION

During the generation of the *pCAG-eGFP* transgenic mice via pronuclear microinjection, the *Akr1A1* gene was disrupted by the integration of the transgene and thus completely abolished the gene expression (Figure 1). AKR1A1 is involved in the major portion (85%) of the catalysis that converts D-glucuronate to L-gulonate in the synthetic pathway [17, 22]. The *Akr1A1* mRNA expression in the *Akr1A1*eGFP/+ mice exhibited a 50% reduction compared to WT mice (Figure 1C); however, the serum ascorbic acid concentration was the same in the *Akr1A1*eGFP/+ and WT mice but was nearly eliminated in the *Akr1A1*eGFP/eGFP mice (Figure 2A). These results suggest that less than 50% of *Akr1A1* expression in mice is sufficient for ascorbic acid synthesis.

Osteoporosis of the femur was the marked phenotype of the *Akr1A1* KO mice. The consequences of the osteoporosis included severe trabecular bone loss, decreased BMD, cortical bone thickness and bone marrow cavity enlargement in the distal femur. Moreover, the bone mass of the femoral head and neck in the proximal femur of the *Akr1A1* KO mice was also decreased compared with the *Akr1A1*eGFP/+ and *Akr1A1*eGFP/eGFP mice supplied with ascorbic acid. According to the water intake of the mice (6-12 mL/day) [23] and the ascorbic acid concentration supplied via the drinking water (350 mg/L), the ingested dosage of the mice in the *Akr1A1*^eGFP/eGFP^ + Vit. C group was 2.1-4.2 mg per day, and this dosage was recommended for the maintenance of other ascorbic acid-deficient mouse colonies (*Gulo* KO mice) [24, 25]. Based on the measurement of serum ascorbic acid in the mice, the ascorbic acid level in the *Akr1A1*eGFP/eGFP + Vit. C group was only ~1/4-fold of that in the *Akr1A1*eGFP/+ and WT groups (Figure 2A); however, the ROS levels and the femur structures were identical to the WT and *Akr1A1*eGFP/+ groups (Figure 2D and 2E). This result demonstrated that the ascorbic acid supplement in the present study was sufficient to maintain oxidative stress in the normal range and was also sufficient to rescue the osteoporosis phenotype in the *Akr1A1* KO mice. Moreover, the identical ascorbic acid levels and the architecture of the femurs between the *Akr1A1*eGFP/+ and WT mice indicate that the *Akr1A1*eGFP/+ mice may provide a better control group that avoids the variation between the inbred transgenic colony and the WT mice.

Ascorbic acid is known to be important for the formation and maintenance of bone mass [17, 26], but the detailed mechanisms involved in these processes are still unknown. A previous study showed that the bone formation marker osteocalcin was decreased in *Gulo* KO mice after 3-4 weeks of ascorbic acid withdrawal compared with WT mice [27]. In the present study, similar results were also observed in the *Akr1A1* KO mice in which ascorbic acid was deficient since birth (Figure 6C). However, there was no significant difference in the levels of the bone resorption marker CTX-I between the *Akr1A1*eGFP/eGFP + Vit. C and *Akr1A1*eGFP/+ groups (Figure 6F). These results suggested that the osteoporosis in the *Akr1A1*eGFP/eGFP mice was due to insufficient bone formation possibly due to deficient ascorbic acid production.

Vitamin C has been shown to exert positive effects on differentiating mouse embryonic stem (ES) cell, bone marrow stromal cell line, calvaria-derived cells or osteoblast-like cells to become osteoblasts [28, 29]. In addition, previous studies showed that Vit. C-treated osteoblast-like cells may activate osterix [30] or prolyl hydroxylase domain (PHD) [31] gene transcription at the early stage of osteoblast differentiation and then stimulates initial deposition of collagen followed by induction of specific genes associated with the osteoblast phenotype [32, 33]. Furthermore, Vit. C-treated osteoblasts also up-regulated osteogenic growth factors, including transforming growth factor (TGF)-β, estrogen receptor (ER)-α and osteopontin which stimulated bone formation [34].

In humans, insufficiency of ascorbic acid intake has been known to increase the risk of osteoporosis and fracture [13, 35, 36]. Epidemiological studies have shown that there is an increased prevalence of osteopenia and osteoporosis in premenopausal women from impoverished areas where the people consume an insufficient Vit. C from fruits and vegetables [37]. Moreover, current smoking behavior, which increases the amount of free radicals, was shown to decrease the BMD of the femoral neck in the non-ascorbic acid supplement group [13]. In these cases, inadequate peak bone mass due to malnutrition or other factors that influence bone formation resulted in the development of osteoporosis without the occurrence of accelerated bone loss. In the present study, ascorbic acid administration was able to control the osteoporotic status of the *Akr1A1* KO mice according to ascorbic acid supplementation and withdrawal during development. Therefore, the mice might represent a unique osteoporosis model in which the osteoporosis is caused by insufficient peak bone mass at a young age, which is distinguished from the hypogonadism-induced bone loss that was commonly used as the osteoporosis model for postmenopausal women and elderly people [38].

Vitamin C deficiency increased cancer occurrence and shortened survival in murine model [39] and patients [40]. Previous studies demonstrated that Vit. C supplementation obviously reduced the breast cancer-specific death [41] and prostate cancer occurrence [42]. High-dose Vit. C administration also inhibited the proliferation of cancer cells, and induced tumor cell apoptosis in mouse model *in vitro* and *in vivo* [43]. In addition, Vit. C deficiency increased the progression of murine melanoma and breast cancer cells xenografts in *Gulo*^−/−^ KO mice, while supplementation of Vit. C inhibited metastasis, tumor growth and inflammatory cytokine secretion in tumor-engrafted *Gulo*^−/−^ KO mice [39, 44]. Similar to *Gulo*^−/−^ KO mice, aldo-keto *reductase*s *(*AKRs) knockout mice also become scorbutic without Vit. C supplementation, but this model has not yet been studied in cancer research [39]. Currently, the roles of AKRs in tumorigenesis have been reported, such as Goh et al. [45] found that AKR1A1 protein expression was significantly decreased in hepatocellular carcinoma.

In summary, *Akr1A1* KO mice presented with a reduction in ascorbic acid production and an increased level of ROS in the serum and, consequently, showed insufficient trabecular and cortical bone mass of the femur at a young age. Through *in vivo* or *ex vivo* micro-CT analysis, the knockout mice may serve as a useful model to mimic the development of osteoporosis due to sub-optimal bone growth rather than accelerated bone resorption during childhood and adolescence.

## MATERIALS AND METHODS

### Animals

The animals used in this study were approved by the Institutional Animal Care and Use Committee (IACUC No.: 103-97) of National Chung Hsing University. The male and female *Akr1A1* KO mice (*Akr1A1*eGFP/eGFP) that contain the genetic background of the CD-1 mouse strain were generated by pronuclear microinjection [46], and a *pCAG-eGFP* vector was double digested with *Sal*I and *Hind*III and used as a transgene. The *Akr1A1* gene knockout was the result of an integration of the transgene and the results were confirmed using compatible ends ligation inverse-PCR (CELI-PCR) [15], quantitative RT-PCR (Q-PCR) and immunohistochemistry (IHC). The originally produced male and female founder mice (F0) were bred with wild-type (WT) CD-1 mice for at least 2 generations to avoid multiple integrations of the transgene [47].

The female mice in the following biochemical and osteoporotic analyses were divided into 4 groups, *Akr1A1*eGFP/eGFP, *Akr1A1*eGFP/eGFP + Vit. C, *Akr1A1*eGFP/+ and WT. In the *Akr1A1*eGFP/eGFP + Vit. C group, the mice were supplemented with ascorbic acid (350 mg/L; Sigma-Aldrich, St. Louis, MO, USA) in drinking water since the day of birth, and the fresh prepared ascorbic acid containing water was changed 2 times a week. All of the mice were fed with a regular diet lacking ascorbic acid until 12 weeks of age. At the end of the 12th week, the mice were sacrificed, and the blood serums and femoral bones were isolated for later analysis.

### Immunohistochemistry (IHC)

The liver tissues, which were obtained from anesthetized WT or *Akr1A1*eGFP/eGFP mice, were immediately fixed using 10% formalin for at least 16 h. The 3 μm paraffin-embedded sections of fixed liver were used for immunostaining of the AKR1A1 protein. Briefly, the sections were deparaffinized by 100% xylene and rehydrated by a series of ethanol washes (100%, 90%, 80% and 60%), and the antigens were then retrieved using retrieval buffer (10 mM sodium citrate, 0.05% NP-40, pH 6.0) at 95 °C for 30 min. The sections were incubated with 3% H_2_O_2_ for 10 min to block the endogenous peroxidase activity, and horse serum for 30 min and then incubated with polyclonal rabbit anti-AKR1A1 (1:500; Sigma-Aldrich) at 4 °C for 16 h. The AKR1A1 protein signal was amplified using the Elite ABC Kit (Vector Labs., Burlingame, CA, USA). Finally, the sections were stained with 3,3’-diaminobenzidine (DAB) and counterstained with hematoxylin [48, 49]. The images of the sections were observed and captured using the Zeiss Axio Scope.A1 microscope (Zeiss, Germany).

### Quantitative RT-PCR (Q-PCR)

Total RNA was isolated from the *Akr1A1*eGFP/eGFP, *Akr1A1*eGFP/+, and WT mouse livers using TRIzol reagent (Invitrogen Corp., Grand Island, NY, USA) according to the manufacturer's instructions. The 1 μg of the total RNA was used for the first-strand cDNA synthesis by the ImProm-II Reverse Transcription System (Promega, Madison, WI, USA). The *Akr1A1* mRNA expressions were analyzed using the relative standard curve method, and the mRNA expressions of *β-actin* gene were used as an internal control [50]. The reactions were performed with the *Akr1A1* forward primer 5’-CAACTGGAGTATTTGGACCTC-3’ and reverse primer 5’-GACATCATCAATCTGCCGAC-3’. The PCR conditions were 95 °C for 2 min, 40 cycles of 95 °C for 30 sec, 56 °C for 30 sec and 72 °C for 20 sec, and followed by a 65 °C to 95 °C melting step.

### The quantitative measurement of ascorbic acid, ROS, osteocalcin and CTX-I in serum

After anesthetization, the mouse blood was isolated by cardiac puncture, then the blood was centrifuged by 3000 rpm for 10 min to separate the serum from blood cells, and stored at -80 °C until use. The concentration of ascorbic acid, ROS, osteocalcin [51] and CTX-I [52] in the serum were measured by Ascorbic Acid Assay Kit (Abcam, Cambridge, MA, USA), OxiSelect^TM^ *In Vitro* ROS/RNS Assay Kit (Cell Biolabs, Inc., San Diego, CA, USA) and enzyme-linked immunosorbent assay (ELISA) (Cloud-Clone Corp., Houston, TX, USA), respectively, according to the instruction manuals.

### Micro-CT analysis of femur

The femurs from 12-week-old mice of the experimental groups were fixed in 10% formalin for 24 hours, and then X-ray micro-CT was performed by Skyscan 1076 machine (Bruker microCT, Kontich, Belgium) with 9 μm resolution [53]. The raw data was analyzed according to the standard procedure [54]. Briefly, the scanning results were first reconstructed into 2D model by NRecon software (Bruker microCT). Then, according to the region of interesting (ROI) selection of the femurs, the characters of the trabecular and cortical bone were analyzed by CTAn software (Bruker microCT), and 3D model of the ROI region were constructed by CTvol software (Bruker microCT). The ROI of trabecular bone was selected for 1.8 mm thick that was started from the epiphysis plate in distal femur (Figure 3A), and the microarchitecture of the trabecular bones were analyzed. Trabecular bone volume (BV/TV) was calculated using bone volume (BV) and total tissue volume (TV). Mean trabecular thickness (Tb.Th) was determined based on the local thickness. Trabecular separation (Tb.Sp) and trabecular number (Tb.N) were estimated using the plate model. The ROI of the cortical bone were selected for 0.9 mm thick followed by the ROI of trabecular bone (Figure 3A), and the characters of the cortical bones were analyzed, including: BV/TV, BS / BV, BMD and femur width.

### Colony forming unit-osteoblast (CFU-OB) assay

The unattached bone marrow cells obtained from the *Akr1A1*eGFP/eGFP, *Akr1A1*eGFP/eGFP with ascorbic acid supplement and *Akr1A1*eGFP/+ mouse femurs, and the cells were cultured with α-MEM containing 10% FBS, 50 μg/ml L-ascorbic acid and 10 mM β-glycerophosphate in 35 mm culture dish with the density of 2.5 × 10^6^ cells per dish [55]. The medium was changed every 3 days. At the end of the 28th day, the cells were stained with ALP staining kit (Sigma-Aldrich) according to the instruction manual and the images were captured under anatomic microscope.

### *In vitro* osteoclast formation assay

The unattached bone marrow cells obtained from the *Akr1A1*eGFP/eGFP, *Akr1A1*eGFP/eGFP with ascorbic acid supplement and *Akr1A1*eGFP/+ mouse femurs, and the cells were cultured with α-MEM containing 10% FBS, 50 ng/ml RNAKL and 25 ng/ml M-CSF in 35 mm culture dish with the density of 2.5 × 10^6^ cells per dish [56]. The medium was changed every 3 days. At the end of the 14th day, the cells were stained with TRAP staining kit (Sigma-Aldrich) according to the instruction manual and the images were captured under anatomic microscope.

### Statistical analysis

All data were presented as the mean ± standard error of mean (SE). The Student's *t*-test and the one-way ANOVA with Duncan's new multiple range test were used for the comparisons of two and multiple groups, respectively. A *P*-value < 0.05 was considered significant.

## SUPPLEMENTARY MATERIALS FIGURES AND TABLES


