# Supplementary material for: A novel osteoporosis model with ascorbic acid deficiency in Akr1A1 gene knockout mice
Source: Oncotarget. 2017 Jan 2;8(5):7357–69. doi: 10.18632/oncotarget.14458 (PMC5352327; doi:10.18632/oncotarget.14458)
Supplement: Supplementary file 1 [file oncotarget-08-7357-s001.pdf]

A novel osteoporosis model with ascorbic acid deficiency in *Akr1A1* gene knockout mice

Cheng-Wei Lai[1,2,*], Hsiao-Ling Chen[3,*], Min-Yu Tu[1,4,5], Wei-Yu Lin[1,2], Theresa Röhrig[1,2], Shang-Hsun Yang[6], Ying-Wei Lan[1,7], Kowit-Yu Chong[7,8] and Chuan-Mu Chen[1,2,9]

[1] Department of Life Sciences, National Chung Hsing University, Taichung, Taiwan

[2] Agricultural Biotechnology Center, National Chung Hsing University, Taichung, Taiwan

[3] Department of Bioresources, Da-Yeh University, Changhua, Taiwan

[4] Department of Orthopaedic Surgery, Taichung Armed Forces General Hospital, Taichung, Taiwan and National Defense Medical Center, Taipei, Taiwan

[5] Department of Biomedical Engineering, Hungkuang University, Taichung, Taiwan

[6] Department of Physiology, and Institute of Basic Medical Sciences, National Cheng Kung University, Tainan, Taiwan

[7] Department of Medical Biotechnology and Laboratory Science, College of Medicine, Chang Gung University, Tao-Yuan, Taiwan

[8] Department of Thoracic Medicine, Chang Gung Memorial Hospital at Linkou, Tao-Yuan, Taiwan

[9] Rong-Hsing Translational Medicine Center, and iEGG Center, National Chung Hsing University, Taichung, Taiwan

[*] These authors have contributed equally to this study

Correspondence to: *Chuan-Mu Chen,* ***email***: *chchen1@dragon.nchu.edu.tw*

Correspondence to: *Kowit-Yu Chong,* ***email***: *kchong@mail.cgu.edu.tw*

Keywords: osteoporosis; Akr1A1 gene; ascorbic acid; knockout mice; micro-CT imaging; trabecular bone; cortical bone; Pathology Section

Received: *August 13, 2016* ***Accepted***: *December 07, 2016* ***Published***: *January 02, 2017*

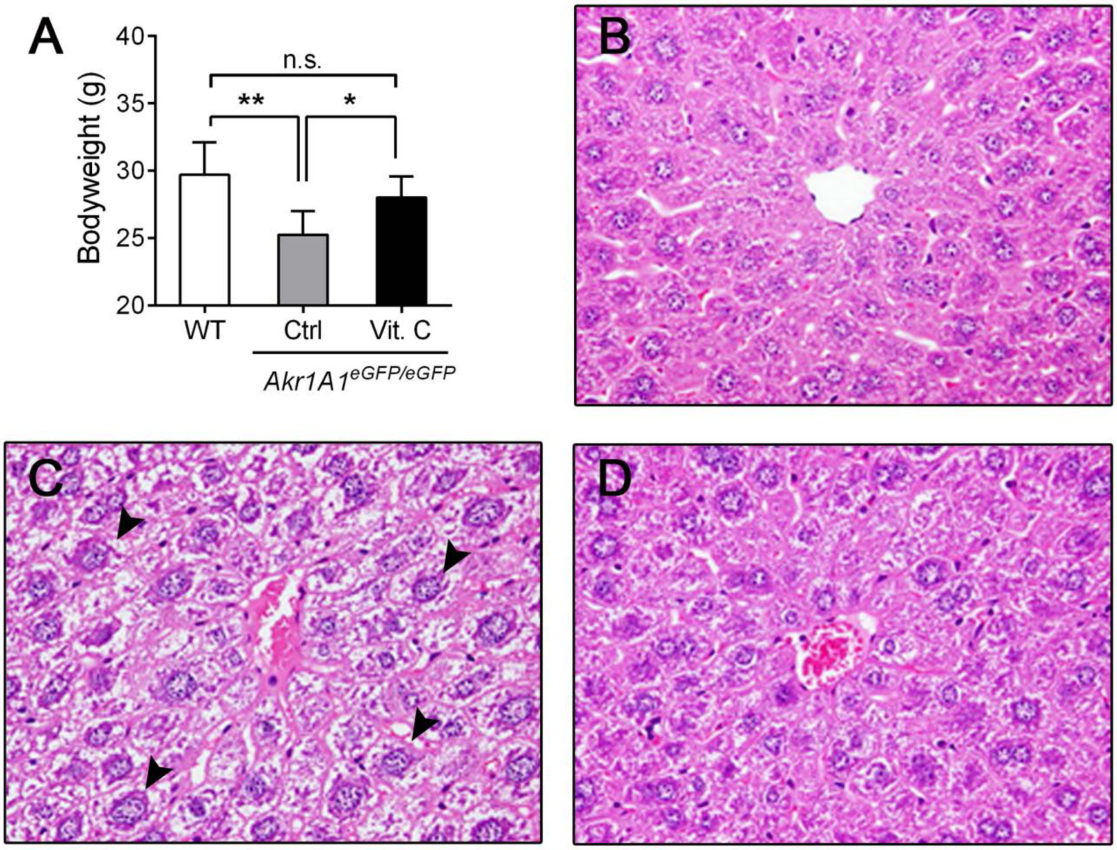

Supplementary Figure S1. Body weight and liver pathological changes of Akr1A1 KO mice. (A) Body weights were measured at the end of treatment (12 weeks old). Liver tissues were collected for sectioning and H&E staining. The representative liver images are shown as (B) WT mice, (C) Akr1A1 KO mice, and (D) Akr1A1 KO mice + Vit C. Solid black arrowheads represented the aging of hepatocytes. Original magnification, 400x.
